# Semi-Solid Pharmaceutical Formulations for the Delivery of Papain Nanoparticles

**DOI:** 10.3390/pharmaceutics12121170

**Published:** 2020-12-01

**Authors:** Caroline S. A. de Lima, Justine P. R. O. Varca, Kamila M. Nogueira, Gabriela N. Fazolin, Lucas F. de Freitas, Eliseu W. de Souza, Ademar B. Lugão, Gustavo. H. C. Varca

**Affiliations:** 1 Nuclear and Energy Research Institute, University of São Paulo, São Paulo 05508-000, Brazil; justinepaula@usp.br (J.P.R.O.V.); kmnogueira@usp.br (K.M.N.); gabrielafazolin@usp.br (G.N.F.); lucasfreitas@usp.br (L.F.d.F.); ablugao@gmail.com (A.B.L.); 2 Department of Polymers, Technology College (Fatec), São Paulo 03694-000, Brazil; eliseupolimeros2@gmail.com

**Keywords:** nanopapain, semi-solid formulation, therapeutic enzymes, gel, nanoparticles

## Abstract

Papain is a therapeutic enzyme with restricted applications due to associated allergenic reactions. Papain nanoparticles have shown to be safe for biomedical use, although a method for proper drug loading and release remains to be developed. Thus, the objective of this work was to develop and assess the stability of papain nanoparticles in a prototype semi-solid formulation suitable for dermatological or topical administrations. Papain nanoparticles of 7.0 ± 0.1 nm were synthesized and loaded into carboxymethylcellulose- and poly(vinyl alcohol)-based gels. The formulations were then assayed for preliminary stability, enzyme activity, cytotoxicity studies, and characterized according to their microstructures and protein distribution. The formulations were suitable for papain nanoparticle loading and provided a stable environment for the nanoparticles. The enzyme distribution along the gel matrix was homogeneous for all the formulations, and the proteolytic activity was preserved after the gel preparation. Both gels presented a slow release of the papain nanoparticles for four days. Cell viability assays revealed no potential cytotoxicity, and the presence of the nanoparticles did not alter the microstructure of the gel. The developed systems presented a potential for biomedical applications, either as drug delivery systems for papain nanoparticles and/or its complexes.

## 1. Introduction

Papain (EC 3.4.22.2) is an enzyme extracted from the papaya latex—*Carica papaya* Linnaeus [1]. It has a double ellipsoidal shape composed of two different conformation domains and has a molecular mass of 23.350 Da [2]. This proteolytic enzyme has several therapeutic properties such as antibacterial [3], antioxidant [4], and antitumoral activities [5]. Bactericide activity of papain has not been found to be related to its proteolytic activity, but to its amidase and esterase activities [3]. Additionally, it has been demonstrated that papain can protect cells against H_2_O_2_-induced damage [4].

Regarding cancer treatment, cysteine proteinases extracted from plants are likely to affect the balance between proteinases and antiproteinases, which interferes in the tumor metastasis and also interacts with the cytokine network promoting immunosuppression and modulation of the tumor growth [5].

Müller et al. [6] studied the antitumor effect of papain and bromelain in human cholangiocarcinoma cell lines. They demonstrated that both enzymes were able to inhibit NFκB/AMPK signaling and their downstream signaling proteins, impairing tumor growth. Their results showed that the treatment-induced apoptosis and, therefore, the plant extracts were cytotoxic to the cancer cells.

Although enzymes have great potential for pharmaceuticals and cosmetic applications, their use is limited due to their low stability in unusual environments (environments where they are not naturally found), as they tend to be sensitive to temperature and pH changes, as well as to the presence of other components in a cosmetic formulation, which impacts the biological activity of enzymes over a relatively short period [7,8,9]. This reinforces the interest and relevance of obtaining more stable and “resistant” molecules/particles and/or a properly designed pharmaceutical form. Additionally, the toxic effects and allergenic reactions have led to a restriction imposed by the FDA regarding the use of papain and papain-derived products for topical use [10]. The development of enzyme nanoparticles represents an alternative to overcome such problems [11]. For instance, papain nanoparticles previously reported by Varca and co-workers [12,13,14] were hypothesized to have better biopharmaceutical properties—such as cell-specific targeting and improved drug loading capacity—than their bulk counterparts and may be a possible solution for cosmetic or pharmaceutical formulations.

When a formulation is administrated on the skin, the goal is to have a topical treatment or a transdermal effect. In the first case, the formulation drug will act on the skin, and the transdermal effect is supposed to go through the skin layers and get to the systemic circulation. Either way, it is paramount that the formulation crosses the stratum corneum—the outer layer of the skin. One of the strategies that have been explored is to use penetration enhancers—such as papain—that can go through all the skin layers [15]. In addition, papain has been encapsulated in polymer and liposome nanoparticles for its use as a drug. The enzyme showed great results in treating hypertrophic scars [16,17].

On the other hand, the development of pharmaceutical forms using polymeric materials has been explored for several applications, covering areas such as tissue engineering, wound treatment bandages, controlled drug delivery systems, hygiene products, contact lenses, among others [18].

Sodium carboxymethylcellulose (CMC) is a natural polymer obtained from cellulose, with a molar mass between 90,000 and 200,000 g mol^−1^ [19]. CMC has properties of great interest for medical applications, such as biocompatibility, biodegradability, low cost, and high swelling capacity. When applied for wounds and burns treatment, for instance, this polymer provides a suitable environment for the formation of the extracellular matrix and re-epithelialization of the treatment site [19,20].

Poly(vinyl alcohol) (PVA) is a bioadhesive, biodegradable, and water-soluble polymer with a crystalline structure [21]. Bioadhesive systems are very interesting for drug delivery because they provide superior contact time of the drug with the biological tissue, enhancing drug absorption and bioavailability [22,23]. Recently, PVA nanofibers loaded with the peptide dimer A3-APO, which accelerates wound healing and prevents wound infections, were produced. In this study, it was observed that the developed drug delivery system required just one-tenth of the amount of A3-APO usually administrated intramuscularly to present the same bactericidal effect [24]. To improve its biological performance in the area of tissue engineering, PVA is often modified or combined with other molecules [25]. CMC and PVA are frequently combined to load and deliver drugs. Several studies are available in the literature [26,27].

Thus, in this work, we sought the development of a semi-solid pharmaceutical form composed of CMC and PVA gel for the loading of papain nanoparticles for potential dermatological and topical applications, such as the treatment of tumors and wounds. The specific aim was to obtain an effective pharmaceutical form for the use of papain nanoparticles as an active ingredient or adjuvant, while, on the other hand, the use of the nanostructured enzyme aims, among other aspects, to allow papain usage with fewer allergenic reactions and side effects, among other biopharmaceutical advantages shared by the nanoparticulated form.

## 2. Materials and Methods

### 2.1. Materials

Carboxymethylcellulose, poly(vinyl alcohol) (98% hydrolyzed), L-cysteine hydrochloride monohydrate, dimethylsulfoxide, ethanol, sodium hydroxide, acetic acid, ethylenediaminetetraacetic acid, monosodium phosphate, and heptahydrate disodium phosphate were purchased from LabSynth (Diadema, Brazil). Papain 30.000 USP-U/mg was acquired from Merck (Darmstadt, Germany) and Nα-benzoyl-DL-arginine-p-nitroanilide hydrochloride from Sigma-Aldrich (San Luis, MO, USA). All reagents were of analytical grade.

### 2.2. Methods

#### 2.2.1. Nanoparticles Synthesis

Papain nanoparticles were synthesized by diluting in an ice bath, 10 mg mL^−1^ of papain in phosphate buffer (50 mM), followed by the addition of ethanol to reach 20% (*v*/*v*). The samples were then exposed to a γ-radiation from a ^60^Co source at 10 kGy, with a dose rate of 5 kGy h^−1^, under refrigeration, on a Multipurpose Irradiator [28]. Native papain was used as a control and, therefore, was handled and assayed under the same conditions as the nanoparticles.

#### 2.2.2. Development of Semi-Solid Formulations

To prepare the semi-solid formulations, 1 g of PVA was solubilized in 100 mL of Milli-Q H_2_O in a water bath at 90 °C under magnetic stirring and 2 or 3 g CMC were solubilized in 60 mL of water under mechanical stirring at room temperature, separately. As for gel A, 20 mL of the PVA was added to the 2 g CMC solution, followed by the addition of 20 mL of glycerin. In the end, PVA concentration was 0.002 g mL^−1^ (0.2%) and CMC was 0.02 g mL^−1^ (2%). The mixture remained under mechanical stirring (200 rpm) until complete homogenization. The gel was then stored at room temperature for 24 h prior to the addition of the enzyme. The same procedure was repeated to prepare gel B, but using a 3 g CMC gel instead, to reach the final concentrations of 0.002 g mL^−1^ (0.2%) PVA and 0.03 g mL^−1^ (3%) CMC.

#### 2.2.3. Papain Loading

Papain loading was performed by the addition of a papain nanoparticle suspension into the gel at room temperature to reach a final papain concentration of 0.2% (*w*/*v*). The formulation was homogenized carefully with a plastic spatula and stored at 4 °C for stabilization. Native papain solution was used as a control. Therefore, 4 formulations were obtained: gel A containing papain nanoparticles, gel B containing papain nanoparticles, gel A containing native papain (control), and gel B containing native papain (control).

#### 2.2.4. Characterization

##### Nanoparticles Size

The size distribution of papain nanoparticles was evaluated by dynamic light scattering (DLS) on a Zetasizer Nano ZS equipment (Malvern, UK) using 10 runs of 10 s with no interval for each sample, under a temperature of 20 °C and angle of 173°. The test was performed following ISO 22412:2017 [29] using a nanoparticle suspension of 4 mg mL^−1^ (diluted in deionized water).

##### Nanoparticles Crosslinking

The crosslinking level was accessed through bityrosine formation. The samples were filtered using a syringe filter (0.45 µm) and diluted in 50 mM phosphate buffer (pH 7.2) until reaching an absorbance of 0.2–0.4 in the UV spectrum at 280 nm. The fluorescence emission spectrum of bityrosine was then verified using excitation wavelength (λ_Ex_) at 350 nm and scanning the emission length (λ_Em_) ranging from 375 to 500 nm, with excitation and emission bandwidths of 9 and 15 nm. This assay was performed in SpectraMax i3 Multi-Mode (Molecular Devices, San José, CA, USA) [14].

##### Stability Assessment

The gels were stored in lidded glass flasks, subjected to extreme temperature variation cycles (oven at 45 ± 2 °C for 24 h and freezer at −20 ± 2 °C for 24 h), and finally centrifuged at 3000 rpm for 5 min. This whole process was repeated six times (a total of 12 days). After the thermal cycles, the gels were evaluated for pH and organoleptic properties. The test was carried out with the formulations in the absence and presence of nanopapain and native papain. The pH of all formulations (gels with and without papain) was measured with a pH electrode, SevenCompact model (Metler Toledo, Columbus, OH, USA) after diluting them to 5% (*v*/*v*) in neutralized water. Regarding the organoleptic properties, the samples were checked for changes in odor, appearance, and color upon each cycle. This assay was performed according to the Brazilian National Health Surveillance Agency—ANVISA—guidelines [30].

##### Scanning Electron Microscopy

All formulations were previously frozen with dry ice and then lyophilized in a freeze dryer equipment (Solab, Piracicaba, Brazil). Once dried, the samples were cut into slices of 0.5 × 0.5 cm and placed on a stub. The images were obtained without sputter coating on a HITACHI TM3000 (Chiyoda-ju, Tokyo, Japan) scanning electron microscope (SEM), with 30 nm resolution and magnification of 500 times.

##### Protein Distribution

Bradford reagent was prepared using Coomassie Blue dye, by diluting 100 mg in 50 mL of 95% ethanol. To this solution, 100 mL of 85% phosphoric acid was added and the volume was made up to 1 L [31]. In total, 500 µL of Bradford reagent were added and homogenized to 1 g sample of each formulation and allowed to react for 5 min.

##### Proteolytic Activity in the Gel Formulation

Nanoparticle proteolytic activity was quantified using Na-benzoyl-DL-arginine-p-nitroanilide Hydrochloride (BAPNA) as a substrate, according to the protocol from Ferraz and co-authors [32].

The formulations were diluted to 5% (*v*/*v*) (in neutral water), incubated in a water bath at 40 °C with the substrate. The reactions were stopped by using acetic acid (10%) at 0, 15, 30, and 45 min, and the absorbance was read at λ = 405 nm in a SpectraMax i3 Multi-Mode (Molecular Devices, San José, CA, USA). Native papain was used as control. The activity of the isolated native enzyme and nanoparticles were used as a reference for the relative activity calculations. This assay was carried out in triplicates.

##### Papain Release

Papain release was assessed by incubating 1 g of each gel in 15 mL falcon tubes containing 9 mL of Phosphate buffered saline (PBS) under mechanical agitation of 150 rpm and at 37 °C. Absorbance measurements were performed using 200 µL aliquots (λ = 280 nm) at defined time intervals between 0 and 96 h in a SpectraMax i3 Multi-Mode (Molecular Devices, USA). After evaluation, the aliquots were returned to the tubes. As positive controls, native papain and nanopapain were used. The assay was carried out in triplicates.

##### Cytotoxicity

NIH 3T3 cells were seeded in 96-well plates (10^4^ cells per well) and incubated with a mixture of 50 µL of culture medium and 50 µL of the gel diluted in deionized water (50%, *v*/*v*) for 24 h. Then, 25 µL of MTS (3-(4,5-dimethylthiazole-2-yl)-5-(3-carboxymethoxyphenyl)-2-(4-sulfophenyl)-2H-tetrazolium) solution was added to each well and, after 3 h of incubation (at 37 °C, humid atmosphere with 5% of CO_2_), the absorbance of the plates at 490 nm was assessed with a microplate reader (Spectramax i3 Multi-Mode—Molecular Devices, USA).

The results were compared with positive controls (cells incubated with 100 µL of 100% dimethyl sulfoxide) and negative controls (cells incubated with culture medium containing PBS instead of gel samples). Each sample, including controls, was tested in 8 wells of the plate, in three different plates. For this test, only the polymeric matrices were used. The data means of each group were compared with one another using Student’s t-test, considering *p*-value < 0.05.

## 3. Results and Discussion

### 3.1. Nanoparticles

Papain nanoparticles presented an average size of 7.0 ± 0.1 nm (Table 1), as revealed by dynamic light scattering. The increased size of nanopapain compared to the native enzyme suggests a change in its conformation. This might be due to the nanoparticle formation process that occurs through crosslinking between tyrosine residues (bityrosine formation) under irradiation, among other intramolecular interactions, in a controlled manner in the presence of ethanol [13,28]. The enzymatic activity of the nanoparticles was evaluated, and it was observed that 11.5% were lost during the synthesis procedure involving gamma irradiation.

**Figure 1:**
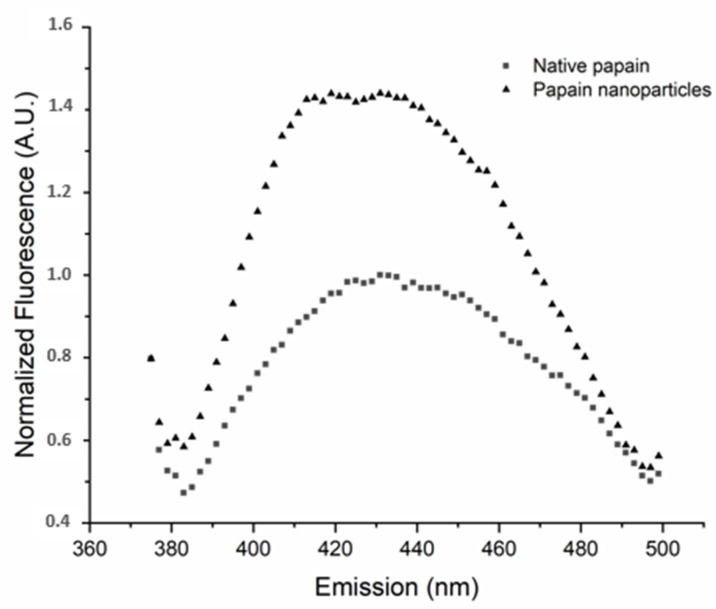
Bityrosine fluorescence spectra of native papain and papain nanoparticles obtained using excitation wavelength (λ Ex) of 350 nm and emission wavelength (λEm) scanning ranging from 350 to 500 nm, with excitation and emission bandwidths of 9 and 15 nm. (A.U. = arbitrary unit).

Bityrosine levels (Figure 1) were studied using fluorescence to verify the formation of crosslinks. The results showed that, in accordance with what was previously observed by our group [13], the bityrosine signal in the nanopapain samples was around 30% higher than the signal in the native papain sample. Fazolin et al. [28] demonstrated that these crosslinks generated in the nanoparticles are rather intramolecular, highlighting no significant changes in the molecular weight of the nanoparticles, and are followed by an increase in bityrosine levels if compared to native papain. Thus, the above-mentioned data highlight the reproducibility of papain nanoparticle synthesis if compared to other reports available in the literature [14,33] and suitable for the development of the study proposed.

### 3.2. Semi-Solid Formulation Stability Assessment

**Figure 2:**
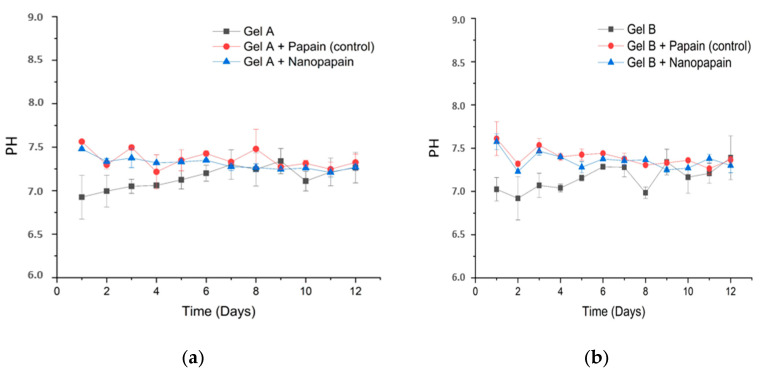
pH measured during stability assessment for the prototype formulations A (**a**) and B (**b**). All measurements were performed by diluting the gels to 5% (*v*/*v*).

All prototype formulations presented a homogeneous and colorless characteristic before and after the addition of the papain, without any signs of instability or precipitation, even upon centrifugation. Although the percentage difference between the two formulations is small, the viscosity was very different. The 3% CMC gel presented a much higher viscosity if compared to the gel using 2% CMC. This would be interesting for different applications and, concerning sterilization steps, a more viscous solution may be interesting in case of some detrimental effects caused by the sterilization process, which often leads to a loss in gel viscosity. The stability of the pharmaceutical form was preliminarily addressed towards assessing the compatibility between the components of the formulation and predicting or anticipating any further incompatibilities. In this study, the samples were subjected to abrupt variations in temperature and high-speed centrifugation. The results obtained during the 12 days of analysis (six cycles) showed that the formulations maintained a neutral pH around 7, as shown in Figure 2a,b.

The organoleptic properties were assessed during the test period, and no signs of destabilization were observed (Table 2). All formulations, even after centrifugation, showed a homogeneous aspect, without precipitation or phase separation. All gels remained translucent and with a mild odor characteristic of glycerin and papain until the 12th day, suggesting that there was no degradation. It is also relevant to highlight that papain is usually not compatible with polymeric matrices due to its enzymatic debridement and amylolytic activity that, in many cases, results in the depolymerization of the matrix [34,35]

**Figure 3:**
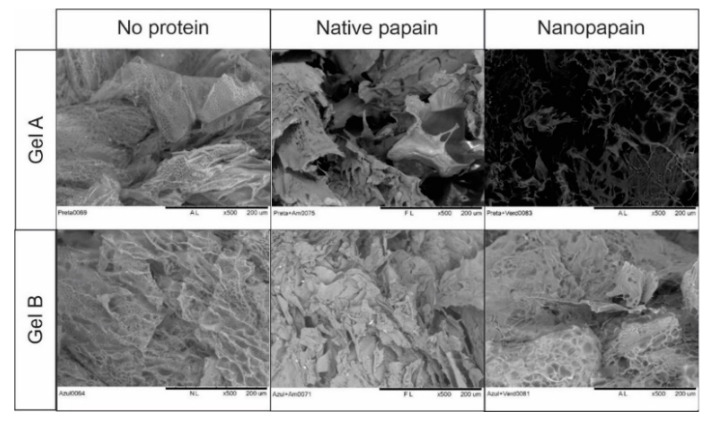
Scanning Electron Microscope (SEM) images of all gel formulations (500× magnification).

The microstructure of each gel was observed by SEM before and after the loading of nanopapain (Figure 3). The SEM was used to track changes in the polymer matrix, as the technique applied in this work does not have a good enough resolution to enable a proper understanding of the modifications related to the enzyme. All the formulations presented a very porous structure, as expected due to the ice crystals formed in the freezing process. The variable pore sizes observed in the images are a consequence of the water content that was removed in the freeze-drying process and are not exactly due to the pores of the 3D gel network, which are much smaller. In addition to this, pore sizes were not controlled in the development of our matrices, as the forces involved in gel formation are rather physical than chemical, and thus small differences may be observed according to the area observed in the sample. The presence of the nanoparticle or native papain did not cause nor lead to any damage to the gel structure, as traceable by SEM. The presence of some crystals due to the semi-crystalline structure of cellulose was also observed and expected to some extent.

**Figure 4:**
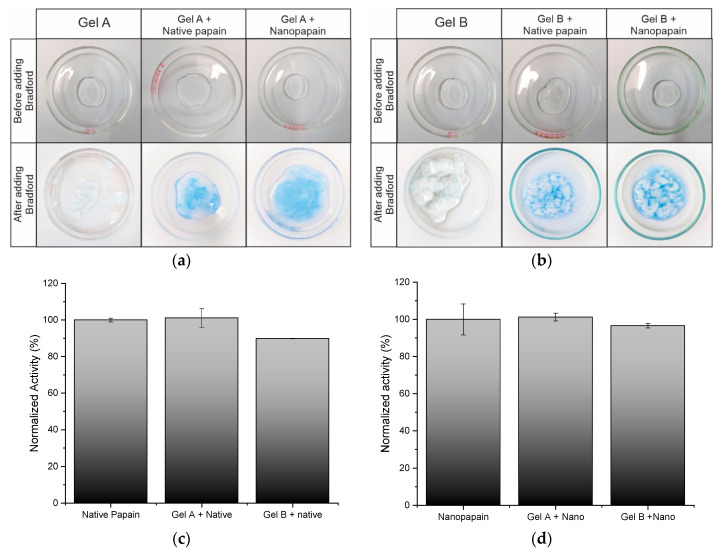
Protein distribution in formulations containing gel A (**a**) and gel B (**b**). Medium proteolytic activity measured of prototype formulations with native papain (**c**) and prototype formulations with papain nanoparticles (**d**) (*n* = 3). Proteolytic activity was determined using Na-benzoyl-DL-arginine-p-nitroanilide Hydrochloride (BAPNA) as a specific substrate at 40 °C and absorbance was assessed at λ = 405 nm.

The papain nanoparticle distribution in the formulations was verified by adapting the Bradford [31] colorimetric method. The technique is typically used for protein quantification, but we attempted to understand the distribution of the enzyme in the gel. As can be seen in the images (Figure 4a,b), Bradford reagent reacted intensely with the papain-containing formulations, compared to a very light color, almost absent, in the gels without papain. It was also noted that the presence of the Bradford reagent destabilized the matrices of gels B (3% CMC and 0.2% PVA), as well as the matrix of gel A without protein (2% CMC and 2% PVA) forming clots in formulations that were previously clearly homogeneous.

In summary, a homogeneous distribution of the protein in the polymer matrix was noted, providing experimental evidence that the formulation was suitable for the loading of both native and papain nanoparticles, which was the main goal of this study. Hereby, we proved that nanopapain featured better stability in the formulation, and also showed that these pharmaceutical forms may be suitable for the administration of the papain nanoparticles and its complexes.

In essence, the preliminary stability tests were performed as a first screening of the proposed formulations, and even though they were promising, long-term stability tests must be addressed at a further stage towards proving and estimating the shelf-life of the formulation or to ensure that it meets the requirements for commercialization. Such tests did not correspond whatsoever to the objectives of the work.

In terms of proteolytic activity, due to the radiolytic process used for the synthesis of the nanoparticles, nanopapain presented a loss of 11.5% of proteolytic activity when compared to the native form of the enzyme (Table 1). This activity loss is expected due to radiation exposure and it is not critical. Such results were in agreement with those obtained by Fazolin et al. [14], considering the parameters adopted in this research.

To calculate the loss of proteolytic activity induced by the gel loading, the activity of the isolated nanoparticles and the isolated native papain were considered to be 100%, respectively, as demonstrated in Figure 4c,d. The nanoparticle loading in formulation A led to no extra loss of activity, while the loading in formulation B caused an activity loss of 3.94%, presenting 96.60% of the nanoparticulated papain activity in suspension. B control formulation containing the native form of the enzyme also presented some loss of proteolytic activity (10.17%) if compared to native enzymes in solution. Such a loss in proteolytic activity was expected due to the processes required for the preparation of the formulations. However, such a loss does not compromise further medical applications, especially as optimization stages will take place before industrial production and commercialization—this is one of the reasons why this study is relevant to the field.

Nevertheless, the formulation produced using gel A ensured no activity loss and, therefore, was more compatible with nanopapain, from a proteolytic activity point of view. It is also relevant to highlight that the nanoparticulated enzyme was slightly less sensitive to the activity loss involved in the formulation stage in the case of gel B.

**Figure 5:**
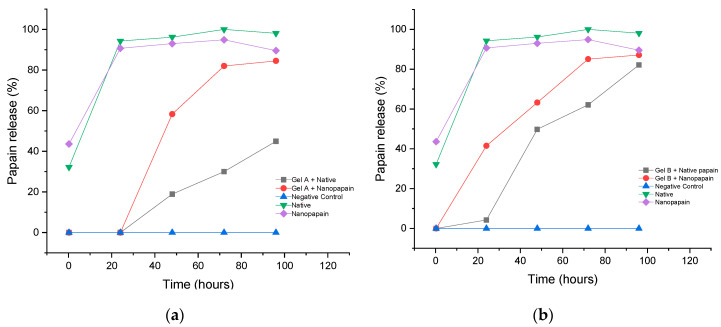
Papain release assessment in gel A (**a**) and gel B (**b**) (λ = 280 nm).

Concerning papain release from the gel matrices, the assay demonstrated a slow release during a period of 96 h (four days) for all formulations (Figure 5). The solubilization process of papain occurs via a solvation process that takes longer than synthetic molecules. It is demonstrated by our native papain control, which reached 100% of solubilization only on the third day of the experiment. Both hydrogels, A and B, started to release nanopapain first then native enzymes in buffer solution. At 48 h, around 60% (58.27% ± 0.14 and 63.25% ± 0.009, for gels A and B, respectively) of the papain nanoparticles were already released and the maximum of release was achieved on the fourth day of the experiment. For native papain, a slower release was observed for gel A, on the fourth day of testing, only 44.90% ± 0.06 were out of the polymer matrix. On the other hand, for gel B, 82.11% ± 0.01 of native papain was released on the fourth day. The differences between native and nanoparticles release from the same matrix are probably related to the nanostructure of the enzyme. Although the bioactivity and other features of native papain were preserved, structural modifications took place as a result of the process and, therefore, may have played a role in impacting the release of the biomolecule upon being entrapped in similar polymeric networks. Properties such as changes in zeta potential and size are examples of changes that may have led or contributed to such a difference.

Although the outline of both gels was similar, gel A presented a slower release profile. This difference in the profile release might have happened because of the addition of papain after the formation of the 3D gel network. We hypothesize that if the enzyme was added to the solution together with the polymer powder, before gel formation, there would be better entrapment of it and, probably, gel B, of higher CMC content, would present a slower release of it. However, this process would most probably cause a bigger impairment to the enzyme, as it would be under mechanical stirring for a longer period. Adding papain after gel formation, in the case of a formulation with a higher CMC content, might have hindered the entrapment of the protein due to the higher viscosity. A previously published study where papain was entrapped in a polyacrylamide matrix crosslinked by radiation showed a different, but also slow, release profile. The researchers observed some burst behavior due to the deposition of the enzyme on the surface of the hydrogel, but not all of the papain content was released within the 50 h of the experiment. It is clear that the structure of the 3D matrix influences the protein release behavior [36].

**Figure 6:**
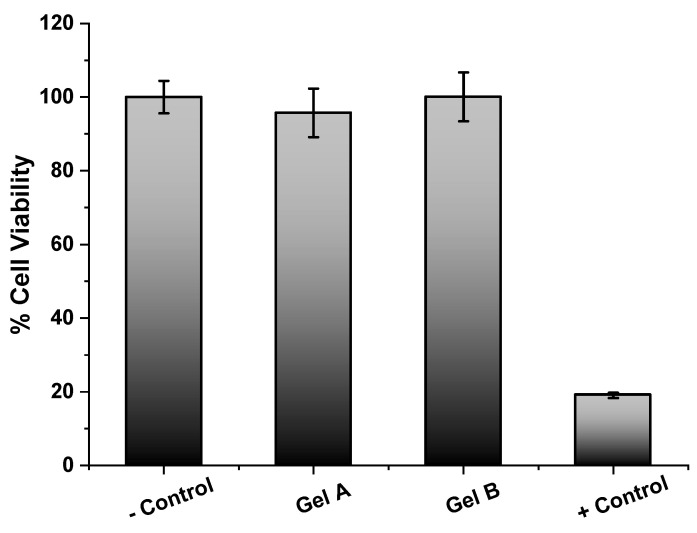
Cytotoxicity assessment of the prototype formulations. MTS dye absorbance was assessed at λ = 490 nm. Control − corresponded to cells without any treatment, whereas Control + corresponded to cells incubated with 100% dimethyl sulfoxide.

Regarding cytotoxicity, the test was performed in formulations A and B in the absence of the enzyme due to the trypsin-like activity of papain, which causes cell detachment and, consequently, a false result of cytotoxicity is obtained in 2D cell culture. The results obtained (Figure 6) revealed that carboxymethylcellulose and poly(vinyl alcohol) matrices did not present any cytotoxicity; for both cases, cell viability was close to 100%. Statistical studies showed a *p*-value < 0.05 and, therefore, confirm the absence of cytotoxicity in the samples. 

## 4. Conclusions

The main objective of this work was the development and assessment of the stability of prototype semi-solid formulations suitable for loading nanopapain for dermatological or topical administration considering the biopharmaceutical advantages of the nanoparticulated papain form. The CMC and PVA gel developed in this work presented a very favorable environment for the enzyme in both forms—native and nanoparticulated, as the proteolytic activity was preserved in both formulations after loading into the polymer matrix. Additionally, our findings suggest that the nanoparticulated form was found to be slightly more stable in the formulations than the native form of the enzyme, as observed by the minimum proteolytic changes. The results also demonstrated that the polymeric matrices did not show any cytotoxicity and that gel formulation A presented a more homogenous nanoparticle distribution and a highly preserved proteolytic activity.

The gel matrix did not suffer any changes in its microstructure in the presence of nanopapain or the native enzyme, maintaining its characteristic porosity. Thus, it is possible to affirm that the developed system holds the potential for biomedical applications, and may be studied either as a vehicle for papain itself or for carrying other drugs through complexation with the nanoparticles, towards improving drug permeation and providing site-specific delivery. In terms of release, both gel formulations were capable of promoting the release of papain nanoparticles, and the native enzyme, despite the idiosyncrasies observed. Further in-depth studies should conduct shelf-life or long-term stability assessments, as well as address advanced biopharmaceutical aspects.

## Figures and Tables

**Table 1:** Sizes measured by dynamic light scattering (DLS) and proteolytic activity.

Sample	Size (d.nm)	Polydispersity Index	Enzymatic Activity
Nanopapain	7.0 ± 0.1	0.34	88.5% ± 7.39
Native papain	3.3 ± 0.4	0.39	100% ± 1.03

Sizes were measured using 10 runs of 10 s with no interval for each sample, under a temperature of 20 °C and an angle of 173°. Tests were made in triplicate.

**Table 2:** Organoleptic properties observed during the stability assessment.

Formulation	Content	Centrifugation	Aspect	Color	Smell
Gel A	0.2% PVA 2% CMC	stable	Homogeneous and translucent	Colorless	No change
Gel A + papain (control)	Gel A + 0.2% native papain	stable	Homogeneous and translucent	Colorless	No change
Gel A + nanopapain	Gel A + 0.2% nanopapain	stable	Homogeneous and translucent	Colorless	No change
Gel B	0.2% PVA 3% CMC	stable	Homogeneous and translucent	Colorless	No change
Gel B + papain (control)	Gel B + 0.2% native papain	stable	Homogeneous and translucent	Colorless	No change
Gel B + nanopapain	Gel B + 0.2% nanopapain	stable	Homogeneous and translucent	Colorless	No change
